# Metabolic Chaos in Kidney Disease: Unraveling Energy Dysregulation

**DOI:** 10.3390/jcm13226772

**Published:** 2024-11-11

**Authors:** Priya Gupta, Saiya Zhu, Yuan Gui, Dong Zhou

**Affiliations:** 1 School of Medicine, University of Connecticut, Farmington, CT 06030, USA; prgupta@uchc.edu; 2 Division of Nephrology, Department of Medicine, School of Medicine, University of Connecticut, Farmington, CT 06030, USA; szhu@uchc.edu (S.Z.); gui@uchc.edu (Y.G.)

**Keywords:** AKI, CKD, energy metabolism, FAO, glycolysis

## Abstract

Background: Acute kidney injury (AKI) and chronic kidney disease (CKD) share a fundamental disruption: metabolic dysfunction. Methods: A literature review was performed to determine the metabolic changes that occur in AKI and CKD as well as potential therapeutic targets related to these changes. Results: In AKI, increased energy demand in proximal tubular epithelial cells drives a shift from fatty acid oxidation (FAO) to glycolysis. Although this shift offers short-term support, it also heightens cellular vulnerability to further injury. As AKI progresses to CKD, metabolic disruption intensifies, with both FAO and glycolysis becoming downregulated, exacerbating cellular damage and fibrosis. These metabolic alterations are governed by shifts in gene expression and protein signaling pathways, which can now be precisely analyzed through advanced omics and histological methods. Conclusions: This review examines these metabolic disturbances and their roles in disease progression, highlighting therapeutic interventions that may restore metabolic balance and enhance kidney function. Many metabolic changes that occur in AKI and CKD can be utilized as therapeutic targets, indicating a need for future studies related to the clinical utility of these therapeutics.

## 1. Introduction

Metabolism encompasses a complex network of chemical reactions and processes essential for life, facilitating the conversion of energy, the synthesis of building blocks for storage, and the elimination of waste products [1]. Within this framework, energy metabolism specifically plays a crucial role in maintaining energy homeostasis by regulating the generation and consumption of energy, primarily in the form of adenosine triphosphate (ATP). This finely tuned system includes diverse biochemical pathways: catabolic pathways break down molecules to release energy, while anabolic pathways synthesize more complex macromolecules from simpler ones. Simultaneously, waste disposal pathways ensure the removal of byproducts resulting from both catabolic and anabolic activities [2].

Key metabolic pathways central to cellular energy production include glycolysis, the tricarboxylic acid (TCA) cycle, glycogen metabolism, and oxidative phosphorylation [3]. Together, these processes ensure a continuous supply of energy required for cellular function and survival. The reliance on specific metabolic pathways can vary significantly across organs—and even among cell types within a single organ—depending on distinct substrate availability and unique metabolic demands [4]. Disruptions in these pathways can lead to insufficient energy production, contributing to disease states such as diabetes, obesity, and cancer.

Recent evidence highlights the significant role of metabolic dysfunction in kidney diseases [5,6,7,8,9,10,11,12], particularly acute kidney injury (AKI) and chronic kidney disease (CKD). The transition from AKI to CKD is especially concerning, as metabolic shifts during this period can exacerbate cellular damage, hinder repair, and accelerate excessive scar tissue formation, known as fibrosis. One prominent feature of this progression is the persistent reduction in fatty acid oxidation (FAO) in kidney cells [7,12,13,14], which deprives them of critical energy sources needed for recovery. FAO is the metabolic process by which fatty acids are broken down in the mitochondria to generate energy, producing acetyl-CoA, nicotinamide-adenine dinucleotide (NADH), and flavin adenine dinucleotide (FADH_2_) as key byproducts. This pathway is essential for maintaining energy balance and supporting cellular functions, especially during periods of fasting or prolonged exercise. Compensatory shifts, like upregulated glycolysis, provide temporary energy relief but also contribute to inflammation and fibrosis, ultimately promoting CKD progression.

Deciphering the complex mechanisms of energy metabolism in renal health and disease is essential for developing targeted therapeutic strategies. By focusing on specific metabolic pathways, there is an opportunity to intervene early in the progression of kidney disease, preserving kidney function and improving patient outcomes. This emerging approach holds promise for innovative therapies that restore metabolic balance, potentially transforming kidney disease treatment and offering patients a new level of protection against disease progression.

## 2. Energy Metabolism in the Kidneys Under Physiological Conditions

The kidneys perform numerous critical functions in the body, including hormone balance, erythropoiesis, blood pressure regulation, fluid balance, and blood filtration, resulting in urine production [15]. These roles, particularly the excretion and reabsorption of nutrients and waste, demand substantial energy. The kidneys receive approximately 20% of the cardiac output and account for around 10% of the body’s oxygen consumption, rendering them highly susceptible to hypoxic injury and diseases when oxygen and energy supply are compromised [16]. Under physiological conditions, the primary sources of energy for the kidneys are FAO and glycolysis. Notably, the proximal tubule of the nephron has a high energy requirement for the active transport of sodium from the bloodstream [17]. This need is reflected in the high density of mitochondria within the proximal tubular epithelial cells (PTECs). The mitochondria of the PTECs predominantly rely on FAO rather than other energy-generating pathways due to its more efficient ATP production, which is thus better able to meet the high energy demand of the PTECs [18].

### 2.1. Fatty Acid Oxidation and the TCA Cycle in the Kidneys

The kidneys, especially the proximal tubule, have a significant energy demand, primarily met through aerobic respiration [19]. The β-oxidation of long-chain fatty acids is a crucial process that generates acetyl-CoA, which subsequently enters the TCA cycle [20]. This metabolic pathway involves numerous enzymes, many of which may become dysregulated in conditions such as AKI and CKD.

Cluster of differentiation 36 (CD36) functions as a transporter, facilitating the entry of long-chain fatty acids into renal tubular cells [21]. The transport of these fatty acids into the mitochondria relies on carnitine palmitoyl-transferase 1 (CPT1), which is the rate-limiting enzyme in FAO [22]. Various factors regulate the expression of these proteins, notably peroxisome proliferator-activated receptors (PPARs) and the PPARγ coactivator-1a (PGC1α), both of which are transcription factors that modulate the gene expression of enzymes involved in fatty acid metabolism [23]. PPARs, including PPARα, PPARγ, and PPARβ/δ, are nuclear hormone receptors that act as transcription factors to influence metabolic processes [24]. PPARα is highly expressed in the renal cortex and primarily localized in proximal tubules [25], while PPARγ is primarily expressed in the distal medullary collecting ducts and PPARβ/δ is expressed in the renal cortex and medulla [26].

AMP-activated protein kinase (AMPK) is another key regulator that promotes FAO while inhibiting fatty acid synthesis. AMPK activates FAO by phosphorylating and deactivating acetyl-CoA carboxylase (ACC) [27], which catalyzes the conversion of acetyl-CoA to malonyl-CoA, a critical rate-limiting step in fatty acid biosynthesis [28]. By enhancing AMPK activity, fatty acid synthesis decreases, promoting energy efficiency.

### 2.2. Glucose and Amino Acid Metabolism in the Kidneys

Glycolysis, the metabolic pathway that converts glucose to pyruvate, occurs primarily in the proximal tubule, albeit at low levels. In anaerobic glycolysis, pyruvate is then converted into lactate in the cytoplasm [29]. While proximal tubular cells express glycolytic enzymes, they typically participate minimally in glucose metabolism [30]. However, they can engage in gluconeogenesis [31]. Other renal cells, such as podocytes, actively partake in anaerobic glycolysis [32]. Renal pericytes, present in the interstitial compartment, also participate in glycolysis as a way of energy generation for the kidney [33]. Interestingly, in a CKD model induced by subtotal nephrectomy, both FAO and glycolytic enzymes were found to be elevated. Proximal tubules in the damaged kidneys demonstrated enhanced functional glycolysis, yet exhibited a decline in mitochondrial respiration, despite an increase in mitochondrial biogenesis. Furthermore, the analysis of the pyruvate dehydrogenase complex pathway revealed a significant suppression of pyruvate dehydrogenase, indicating a reduced availability of acetyl-CoA derived from pyruvate for the citric acid cycle, which is essential for mitochondrial respiration [12].

Under normal conditions, amino acids contribute less significantly to energy production in proximal tubular cells [34]. However, branched-chain amino acids can provide intermediates for the TCA cycle [35], leading to the production of acetyl-CoA and succinyl-CoA [36], thereby supporting energy metabolism in renal physiology. Understanding these metabolic pathways is crucial for addressing kidney health and the implications of metabolic dysfunction in renal diseases.

## 3. Energy Metabolism Dysregulation in AKI

AKI represents a significant public health challenge characterized by a rapid decline in kidney function, typically assessed through increased serum creatinine levels as a surrogate for glomerular filtration rate (GFR). AKI can also be identified by the presence of oliguria [37]. Other diagnostic biomarkers, such as the fractional excretion of sodium, urea nitrogen levels, urine output, and urine microscopy, are also employed to confirm AKI [38]. Histologically, AKI is marked by necrotic cells, the loss of the brush border, and the swelling of PTECs. The condition can arise from various causes, including ischemia and nephrotoxic agents, such as certain chemotherapeutic drugs [39]. Additionally, AKI is often regarded as a complication of surgical procedures and sepsis [40]. Estimates suggest that AKI affects approximately 20% of adult and 25% of pediatric hospitalized patients, highlighting its serious implications [41,42]. Despite the high burden of AKI, there remains a limited understanding of its underlying causes and effective treatment options.

**Figure 1:**
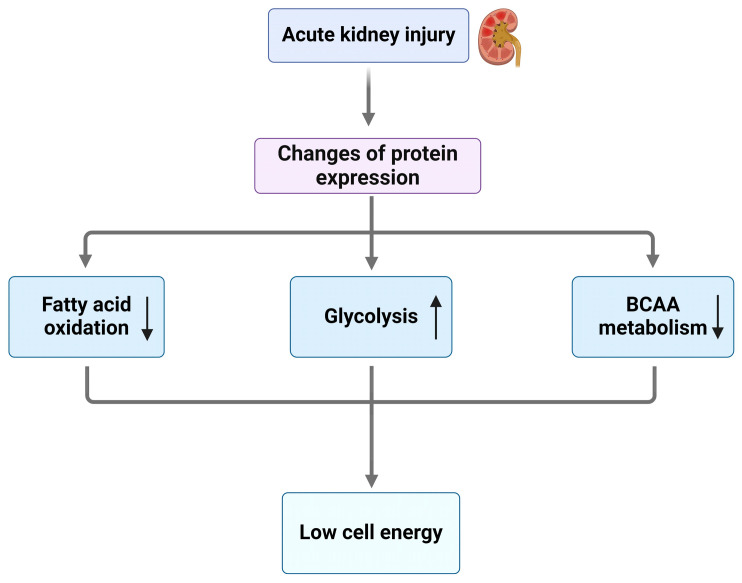
Metabolic shifts in AKI and their impact on cellular energy. Following AKI, key metabolic changes occur as shown in this figure, with an up arrow indicating increased levels after AKI and a down arrow indicating decreased levels after AKI. These changes include decreased FAO, impaired BCAA metabolism, and increased glycolysis. Reduced FAO leads to lipid accumulation, promoting lipotoxicity and mitochondrial dysfunction, while disrupted BCAA metabolism diminishes energy production and elevates toxic byproducts. The shift toward glycolysis compensates for the energy deficit but yields less ATP and leads to lactate buildup, exacerbating acidosis. These changes result in lower cellular energy availability, impairing cell repair and promoting kidney injury progression. AKI, acute kidney injury; FAO, fatty acid oxidation; BCAA, branched-chain amino acid.

Recent hypotheses propose that the development and progression of AKI may be closely linked to alterations in cellular metabolism, particularly within the proximal tubular cells. Due to their high mitochondrial density, PTECs are particularly susceptible to mitochondrial dysfunction, which can significantly impair their function during AKI [43]. These cells are vital for excreting toxins and thus their dysfunction leads to the accumulation of harmful substances within them, further exacerbating injury [44]. The high energy demands of PTECs, primarily driven by their role in the active transport of sodium, render them especially vulnerable to ischemic damage [45]. In response to AKI, FAO—the primary energy source for PTECs—becomes downregulated due to the actions of various regulatory proteins. Although glycolysis is upregulated during AKI, it fails to provide sufficient energy to compensate for the loss of FAO, resulting in the pathological changes characteristic of the condition [30] (Figure 1). Furthermore, vascular endothelial cells within the renal blood vessels sustain damage during AKI [46]. Podocytes, the specialized cells that support glomerular filtration, exhibit injury characterized by foot process fusion and other cellular alterations [47]. Notably, podocytes undergo a metabolic switch that further contributes to the development of AKI [48]. In renal pericytes, a transformation occurs as well. Glycolysis is upregulated in renal pericytes in an attempt to replenish cellular energy stores [33].

### 3.1. Key Genes/Proteins Involved in FAO Defects After AKI

In AKI, metabolism undergoes a switch. Prior to injury, metabolism is predominantly FAO, while afterwards, glycolysis is increased in response to the injury. The switch from FAO to glycolysis is mediated by various cellular changes involving multiple key gene families, including the PPAR, Kruppel-like factor (KLF), and sirtuin gene families.

#### 3.1.1. PPARs Family

PPARs are transcription factors integral to lipid and glucose metabolism [49]. The PPAR family consists of several subtypes, notably PPARγ, which enhances FAO. In AKI, the gene encoding for the farnesoid X receptor (FXR) is downregulated in proximal tubule cells. FXR typically upregulates PPARγ, which promotes FAO, reducing lipid accumulation and tubular cell injury [50]. Additionally, PGC-1α, which activates PPARγ, is decreased in the setting of AKI, leading to suppressed FAO gene expression and increased fibrosis. Consequently, the downregulation of PPARγ and PGC-1α results in diminished FAO levels following AKI [51]. PPARα also plays a crucial role in regulating FAO and is expressed in renal proximal tubular cells. PPARα activates target genes that enhance FAO while also stimulating gluconeogenesis and ketone body synthesis [49]. Studies in mice have demonstrated that lower levels of PPARα correlate with decreased FAO and heightened inflammation [52]. Similar to PPARγ, PPARα also downregulates carnitine, further contributing to reduced FAO following AKI.

#### 3.1.2. The KLF Family

The KLF family of proteins also influences fatty acid oxidation dysregulation in AKI. It is thought that KLF15 works with PPARα to regulate FAO. KLF15 knockout mice exhibit worse destruction of the proximal tubule brush border, greater kidney fibrosis, and increased inflammation [53]. KLF6 has also been associated with worsened kidney injury, as it suppresses BCAA catabolism, thereby blocking an additional energy source for the tricarboxylic acid (TCA) cycle [34]. Collectively, the actions of these KLF proteins contribute to decreased cellular energy in AKI [54].

#### 3.1.3. AMPK

AMPK dysfunction is another contributor to decreased FAO in AKI [55]. Under normal conditions, AMPK phosphorylates and inhibits ACC, promoting FAO and inhibiting fatty acid synthesis [56]. In AKI, the AMPK-mediated phosphorylation of ACC is impaired, resulting in increased fatty acid synthesis and decreased FAO [57]. In mouse models of cisplatin-induced AKI, ACC knockout exacerbates injury, correlating with increased fatty acid synthesis and reduced FAO, as evidenced by elevated urea and creatinine levels, histological changes, and heightened expression of inflammatory markers such as NGAL and IL-6 [21,58]. Thus, ACC inactivation due to AMPK dysfunction is likely contributing to decreased levels of FAO and worsening cellular injury from AKI.

#### 3.1.4. Sirtuins Family

Sirtuins, particularly Sirt5, are involved in regulating FAO. Sirt5 reverses post-translational acylation on enzymes essential for FAO [59]. In ischemia-reperfusion and cisplatin-induced AKI mouse models, Sirt5 knockout resulted in improved kidney function, although FAO levels in mitochondria remained low, suggesting compensation through increased peroxisomal FAO [60]. Sirt1 and Sirt3 also confer protection against AKI [61]. Sirt1 knockdown has been found to correlate with exacerbated inflammation [62]. Sirt3 overexpression promotes autophagy, enhances *p*-AMPK levels, and downregulates *p*-mTOR, alleviating sepsis-induced AKI by reducing tubular cell apoptosis and inflammatory cytokine accumulation [63]. Thus, the downregulation of these sirtuins is associated with worsened kidney function.

#### 3.1.5. Other Genes

AKT and CDK2 also play roles in lipid metabolism regulation. AKT phosphorylates FOXO1, which is hypothesized to be involved in lipid metabolism regulation in tubular epithelial cells. In one study, a FOXO1 inhibitor improved energy metabolism in mice with sepsis-induced AKI [48]. Additionally, the switch from FAO to glycolysis is mediated by the stabilization of hypoxia-inducible factor-1α (HIF-1α) through the Akt/mTORC1/HIF-1α pathway, with mTORC1 being a central player in this transition [64]. Phosphatase and tensin homolog (PTEN) is also involved in this Akt/mTOR pathway. PTEN inhibits the Akt/mTOR pathway, decreasing inflammation. After AKI, PTEN is upregulated, thus exerting a protective effect in renal cells [65].

The interaction among these key genes and proteins illustrates a multifaceted regulatory network that governs FAO in the context of AKI. The downregulation of PPARs, coupled with the actions of KLFs, AMPK, sirtuins, and other metabolic regulators, culminates in a metabolic state characterized by impaired FAO and increased glycolysis. This dysregulation contributes to cellular energy deficits, exacerbating inflammation and renal injury, and underscores the potential for targeting these pathways as therapeutic strategies to mitigate kidney damage and improve outcomes in AKI.

### 3.2. Key Genes/Proteins Involved in Glycolysis in AKI

To compensate for energy loss due to FAO dysregulation [66], renal tubular cells upregulate glycolytic enzymes. Following sepsis-induced AKI, enzymes such as phosphofructokinase (PFKL), which is a rate-limiting enzyme for glycolysis, and pyruvate kinase M (PKM), which catalyzes the final step of glycolysis, are increased. Pyruvate kinase M2 (PKM2) functions similarly to pyruvate kinase in glycolysis in the renal pericytes. However, it also has activity as a transcriptional promoter. PKM2 is similarly activated in AKI. When its activity increases, it facilitates glycolysis directly as pyruvate kinase does [33], but it also plays a role in the metabolic reprogramming that occurs in AKI. This upregulation facilitates glycolytic activation. However, due to decreased oxygen delivery and inefficient oxygen utilization in AKI, glycolysis often occurs anaerobically [67], resulting in pyruvate being converted to lactate instead of acetyl-CoA. Consequently, despite the upregulation of glycolytic enzymes, functional glycolysis remains impaired, failing to fully meet the energy demands of proximal tubular cells [68].

## 4. Energy Metabolism Dysfunction in CKD

Globally, as many as ~25% of AKI cases can progress to become CKD, which poses a significant disease burden [69]. CKD affects approximately 12–14% of the general population and 30–40% of individuals with comorbidities such as diabetes and hypertension [70], often marked by proteinuria persisting over three months. This transition to CKD is driven by renal fibrosis and inflammatory processes that cause cellular death. Post-AKI, nephrons exhibit tubular atrophy and interstitial fibrosis. Maladaptive repair mechanisms, particularly in the tubular epithelium, along with partial epithelial-to-mesenchymal transition, contribute to the development of CKD [71].

**Figure 2:**
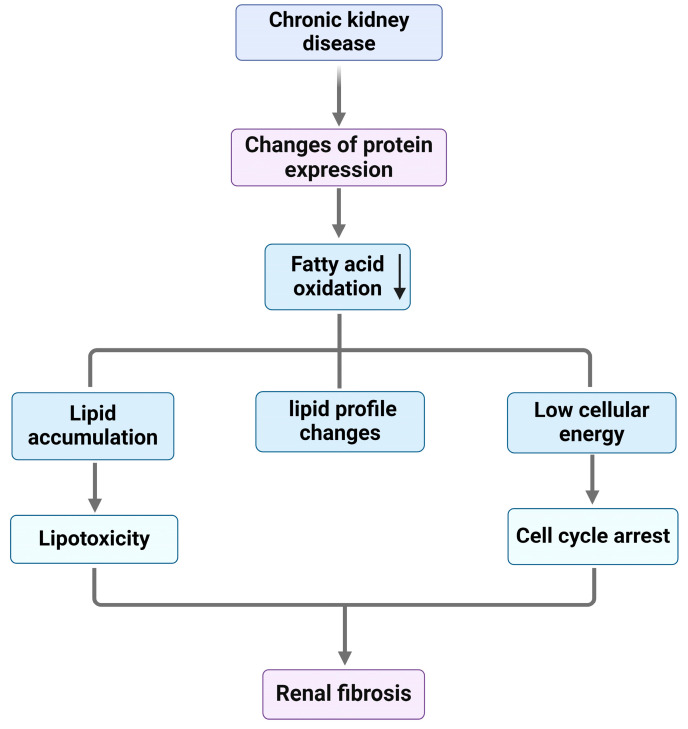
Metabolic alterations driving CKD progression and fibrosis. In CKD, prolonged metabolic changes contribute to energy deficits and promote renal fibrosis. Notable disruptions include lipid accumulation in kidney cells due to impaired FAO and altered lipid metabolism, leading to lipotoxicity and mitochondrial stress. Additionally, changes in lipid profiles disrupt cellular signaling and membrane function. Reduced cellular energy availability causes cell cycle arrest and further hinders the cells’ ability to repair and regenerate. These metabolic changes create a pro-fibrotic environment, driving sustained tissue remodeling, inflammation, and scarring that ultimately progress CKD. CKD, chronic kidney disease; FAO, fatty acid oxidation.

Metabolic changes have been implicated in CKD progression. FAO is reduced in renal tubular epithelial cells during CKD. For example, in an aristolochic acid-induced AKI model, genes associated with FAO were downregulated in both early and late CKD stages [34]. Unlike in AKI, glycolysis is also downregulated in CKD, with genes related to glycolysis and the TCA cycle showing decreased expression [17]. When energy intake surpasses the body’s capacity to store lipids in adipose tissue, ectopic lipid accumulation occurs, leading to lipotoxicity, a detrimental effect on cellular function and viability, and eventually causes renal fibrosis [72]. As CKD progresses, specific lipid profiles also change; one study revealed that worse kidney disease progression correlated with longer triglycerides containing more double bonds [73]. Advanced CKD is associated with increased triglycerides and free fatty acids, with different fatty acids being prevalent at various CKD stages. An accumulation of longer-chain, more toxic lipids in late-stage CKD exacerbates cellular dysfunction and death [74] (Figure 2).

### 4.1. Key Genes/Proteins Involved in FAO in CKD

#### 4.1.1. CPT1a

While metabolic changes drive the progression of CKD, CKD also induces metabolic dysregulation, particularly in lipid metabolism. Genes linked to FAO are downregulated early in CKD and remain low throughout the disease [17]. One of these is CPT1a, an enzyme that transports long-chain fatty acids into the mitochondria for FAO, and which is notably decreased in CKD. Conversely, the upregulation of CPT1a in CKD improves renal function and reduces fibrosis [75]. This process could be mediated by non-PTECs such as fibroblast-derived Calponin 2 [7], through cell–cell communications. CNN2 acts as an actin filament regulatory protein that sends information to neighboring kidney cells. When cells are functioning normally, this process helps maintain cellular architecture. However, in the setting of CKD, this process can contribute to the development of renal fibrosis [7].

#### 4.1.2. PGC1α

Similar to AKI, PGC-1α influences CKD progression, with its role being more pronounced in podocytes than in PTECs [76]. In kidney diseases, particularly diabetic kidney disease, PGC-1α levels in podocytes are reduced. This decrease is correlated with mitochondrial dysfunction and structural and functional kidney damage [77]. PGC-1α regulation may also involve the long-coding mRNA Tug1, with studies suggesting that increased Tug1 levels may restore PGC-1α levels in kidney disease [78].

#### 4.1.3. KLF and Cytokines

As in AKI, KLF15 levels are diminished in CKD. KLF14 has also been implicated in CKD-related changes [79]. KLF14 enhances FAO activity, thereby reducing fibrosis [80]. Cytokines, particularly IL-37, may also contribute to FAO downregulation in CKD. IL-37 is an anti-inflammatory cytokine that is downregulated in diabetic kidney disease. Overexpressing IL-37 has been associated with an upregulation of FAO-related genes and decreased proteinuria and renal fibrosis [51].

In CKD, a similarly intricate network is observed as in AKI, where the downregulation of critical genes such as CPT1a, PGC-1α, and KLFs further impairs FAO, contributing to renal fibrosis and dysfunction. The concurrent upregulation of lipid transporters, like CD36, along with the dysregulation of AMPK, fosters lipid accumulation, aggravating cellular injury through lipotoxicity. These metabolic changes highlight the potential for therapeutic strategies aimed at restoring FAO and reducing lipid accumulation, ultimately improving renal outcomes in CKD.

### 4.2. Key Genes/Proteins Involved in Lipid Accumulation in CKD

In CKD, renal cells exhibit increased lipid uptake due to the upregulation of CD36 [81], a transporter for long-chain fatty acids and low-density lipoprotein (LDL) [82,83]. Enhanced fatty acid uptake promotes lipogenesis. AMPK, which typically inhibits fatty acid biosynthesis in the kidney, may become less active, leading to increased lipid synthesis and accumulation [74]. Excessive lipid accumulation can result in lipotoxicity, characterized by the accumulation of lipids and intermediates in tissues where they are not typically stored [84]. This lipotoxicity occurs due to the accumulation of fatty acyl-CoA, diacylglycerols, and ceramides [85]. This condition can contribute to dyslipidemia-induced kidney injury and ultimately lead to cell death.

## 5. Energy Metabolism Dysfunction in the Transition from AKI to CKD

Although CKD was previously thought to be a distinct condition separate from AKI, recent findings have shown that AKI significantly raises the risk of developing CKD in the months following the initial damage. Even when kidney function seems to fully recover, ATP production often lags behind, leaving cells energetically compromised [86]. The transition from AKI to CKD is marked by a sustained disruption of energy metabolism, particularly the persistent downregulation of FAO. This metabolic dysfunction deprives proximal tubule epithelial cells of the energy needed for proper repair and regeneration. Key enzymes vital to FAO, such as CPT1b and PGC1α, are impaired shortly after injury and remain downregulated for at least 10 days, hampering the cells’ ability to utilize fatty acids for energy. As a result, lipid accumulation ensues, fueling inflammation and fibrosis [87].

**Figure 3:**
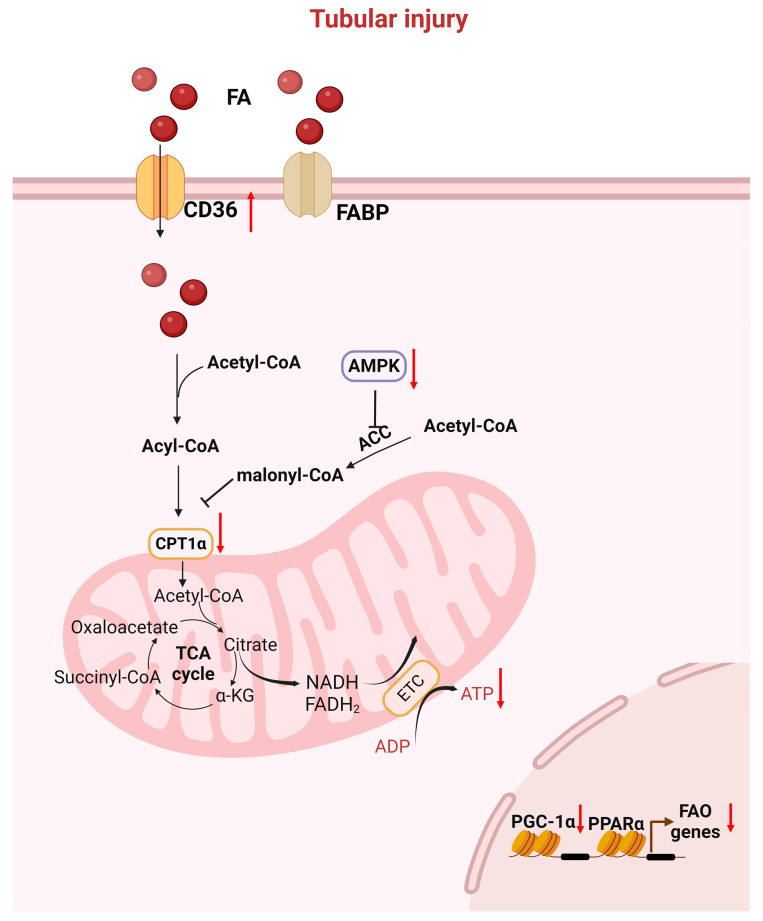
FAO regulation in proximal tubule epithelial cells under normal and AKI–CKD conditions. In a healthy kidney, FAO in proximal tubule epithelial cells is efficiently regulated by essential proteins such as CPT1α, PGC-1α, and AMPK, which support energy homeostasis. Following kidney injury, changes occur as represented by the red arrows in this figure. Up arrows indicate upregulation while down arrows indicate downregulation. Thus, after kidney injury, the upregulation of CD36, a lipid transporter, and downregulation of CPT1α, PGC-1α, and AMPK disrupt FAO, leading to lipid accumulation and mitochondrial dysfunction. This metabolic imbalance contributes to cellular stress, inflammation, and fibrosis, driving AKI progression to CKD. FAO, fatty acid oxidation; CPT1α, carnitine palmitoyltransferase 1α; PGC-1α, peroxisome proliferator-activated receptor gamma coactivator 1α; AMPK, AMP-activated protein kinase.

In an attempt to compensate for this energy shortfall, glycolysis becomes chronically upregulated. Although glycolysis provides a limited energy supply, it paradoxically accelerates fibrosis [88]. Following AKI, tubular epithelial cells are arrested at the G2/M checkpoint and partially transition toward a mesenchymal state, a process that further diminishes their energy-generating capacity and drives fibrosis [17] (Figure 3). Moreover, the upregulation of PKM2 exacerbates this issue. PKM2 is upregulated during AKI to help increase glycolysis in the renal pericytes. While PKM2 initially supports glycolysis during AKI, it also promotes the transformation of renal pericytes into myofibroblasts, the primary producers of fibrotic tissue, thus compounding renal fibrosis [33].

This metabolic imbalance—where FAO remains suppressed, and glycolysis contributes to maladaptive cell states—underlines the chronic dysfunction linking AKI to enduring kidney damage. Addressing this sustained metabolic disruption offers a potential therapeutic avenue to halt or even reverse the progression of AKI to CKD.

## 6. Diagnostic Tools and Therapies for Kidney Disease

In addition to traditional biochemical measurements of serum or urine samples, emerging diagnostic tools—particularly metabolomic markers—offer significant promise for revolutionizing kidney disease management in clinical practice. Metabolomics, a cutting-edge omics technique, is a rapidly advancing field in analyzing metabolites in biological samples, and facilitates the identification of specific biomarkers associated with kidney injury and disease progression [89,90,91,92]. For example, metabolites like 2-hydroxyglutarate and kynurenine have been correlated with acute kidney injury and chronic kidney disease, presenting opportunities for the early detection and monitoring of disease severity [93,94]. Furthermore, metabolic profiling provides valuable insights into the underlying pathophysiological mechanisms, assisting in patient stratification and the development of personalized therapeutic strategies. By integrating these metabolomic markers into clinical workflows, healthcare professionals can enhance diagnostic accuracy, enable timely interventions, and ultimately improve patient outcomes in kidney disease management. Despite its remarkable potential for advancing diagnostics and personalized medicine, metabolomics remains in the early stages of widespread clinical application [95]. While there are notable successes in specific fields, such as oncology and metabolic disorders [89,96,97], its routine implementation for conditions like kidney disease is still limited. Challenges, including the complexity of metabolomic data, the need for standardized methodologies, and the validation of biomarkers across diverse patient populations, must be overcome before metabolomics can achieve full integration into clinical practice [95,98]. Nevertheless, ongoing research is actively investigating its utility, and technological advancements are poised to facilitate its broader adoption in the near future for both diagnosis and treatment.

Currently, renal replacement therapy remains the most widely relied upon treatment for AKI. However, targeting metabolic changes that occur during AKI presents a promising avenue for developing new therapies for both AKI and CKD. A key therapeutic target is the Akt/mTORC/HIF-1α pathway. Hypoxia-inducible factor (HIF) plays a role in shifting metabolism from FAO to glycolysis [99]. When activated, this pathway increases aerobic glycolysis. The pre-stimulation of the Akt/mTORC1/HIF-1α pathway with β-glucan has been shown to improve survival post-AKI [100]. Other potential therapeutics focus on decreasing the activation of this pathway to minimize inflammation. For example, this pathway can also be targeted through PTEN. Upregulating PTEN after AKI has been shown to decrease renal tubular injury. This protein can be targeted in multiple ways. For example, methylating the PTEN promoter site can activate PTEN through regulatory factors, reducing inflammation and fibrosis. mTOR inhibitors, such as rapamycin and everolimus, also decrease the activity of this pathway, potentially decreasing inflammation. However, these targets have been tested in the setting of chemotherapeutic toxicity, not in the setting of AKI, indicating a need for future studies [65]. Additionally, sodium-glucose cotransporter 2 inhibitors (SGLT2is), traditionally used for diabetes and heart failure, target this pathway by limiting glucose reabsorption in the proximal tubule [101]. SGLT2is also prevent renal hypoxia, fibrosis, and inflammation by inhibiting HIF-1α stabilization and increasing FAO via PPARα activation [102,103].

Another key player in kidney disease is AMPK, which is dysregulated in both AKI and CKD. Endurance exercise training (EET) has been found to enhance AMPK activity, reducing fibrosis, inflammation, and lipid deposition [104,105]. AMPK can also be activated by compounds like 5-aminoimidazole-4-carboxamide-1-*β*-D-ribofuranoside (AICAR) [106], which increases PGC-1α levels, providing protection from AKI and reducing inflammation. Other therapeutics, such as metformin and SS-31 can also stimulate AMPK, which would then increase FAO [107]. SS-31, a peptide that penetrates the inner mitochondrial membrane, enhances ATP production and reduces oxidative stress, providing protection in ischemia-reperfusion models [108,109]. Sirtuin (Sirt) activators, such as resveratrol, increase Sirt1 and Sirt3 activity, improving mitochondrial function and survival after AKI [110].

In addition, Cpt1 is a promising target in CKD therapy, which facilitates the mitochondrial uptake of fatty acids. Carnitine supplements have shown potential in increasing fatty acid metabolism [111]. PGC-1α activators like ZLN005 have demonstrated benefits in reducing kidney damage, fibrosis, and lipid accumulation [112]. PGC-1α plays a crucial role in mitochondrial biogenesis and FAO, although its therapeutic window is narrow, with excessive activation leading to complications like albuminuria and azotemia [113,114]. Further strategies include PPAR agonists and CD36 antagonists [115]. Rhein, a rhubarb derivative, activates PPARα, boosting FAO and reducing fibrosis [116]. Ligands such as fibrate and WY14643 have also shown efficacy in improving outcomes in AKI models, particularly following cisplatin or ischemic injury [117]. Fenofibrate has demonstrated the ability to reduce both kidney injury and fibrosis [118].

All of these therapeutic approaches focus on restoring proper cellular metabolism, particularly by increasing fatty acid oxidation. Although these treatments show potential in mitigating the effects of AKI and CKD, further research is required to fully understand their efficacy, safety, and side effects, particularly in patients with comorbidities, especially given that patients with comorbidities are more likely to develop renal disease in the first place. Moreover, additional research is necessary to explore other potential targets implicated in metabolic changes. For example, in theory, blocking PKM2 may reduce the transformation of renal pericytes into myofibroblasts, thus decreasing renal interstitial fibrosis. However, this target has not yet been tested. Considering all involved parts of the metabolic pathways implicated in AKI and CKD as potential therapeutic targets is critical in order to discover which therapeutics are most useful clinically.

Clinically, several ongoing trials and FDA-approved therapies that focus on FAO and glycolysis are promising for treating AKI or CKD. In FAO interventions, two phase 3 clinical trials examine the use of the mitochondrial-targeted peptide Elamipretide or ATP citrate lyase inhibitor Bempedoic Acid in patients with kidney diseases, aiming to enhance mitochondrial function and reduce lipid toxicity. For glycolysis-focused interventions, the DAPA-CKD study demonstrated that dapagliflozin (an SGLT2 inhibitor) significantly reduced the risk of substantial kidney function decline, end-stage renal disease, and renal or cardiovascular death in CKD patients, regardless of diabetes status [119]. In comparison, the EMPEROR-Reduced trial similarly demonstrated that empagliflozin reduced cardiovascular death or heart failure hospitalization in heart failure patients, offering broad benefits in kidney and heart function management [120]. These SGLT2is not only enhance glucose excretion but also modulate metabolic pathways, including glycolysis and FAO. In addition, metformin, a widely used treatment for type 2 diabetes, not only lowers blood glucose levels but also influences metabolic pathways by promoting glycolysis and reducing gluconeogenesis. This dual action is thought to offer protective effects for kidney health [121,122], even in moderate-to-severe CKD [123]. However, a significant caution is its association with lactic acidosis, especially when kidney function is compromised, as metformin can accumulate in the body due to AKI. This buildup may lead to severe acidosis, resulting in gastrointestinal symptoms and, in extreme cases, cardiovascular collapse [124]. Together, these findings suggest that metabolic pathway modulation could improve renal outcomes, but more investigation is needed to refine treatment strategies for kidney disease.

## 7. Conclusions and Perspectives

Metabolic shifts are well-documented across various kidney diseases, where reduced activity through essential pathways leads to critical energy deficits that drive cellular injury, including progressive fibrosis. The impairment of vital processes, such as the electron transport chain, not only limits ATP production but also triggers excessive reactive oxygen species production, which exacerbates cellular damage. In both AKI and CKD, FAO is severely compromised, creating an enduring energy shortfall. During AKI, proximal tubule epithelial cells attempt to offset this deficit by upregulating glycolysis. However, as AKI advances to CKD, even glycolytic pathways fail to maintain energy needs, resulting in an intensified metabolic failure. This sustained disruption in FAO drives fibrosis, accelerates cellular damage, and significantly contributes to CKD progression post-AKI. These metabolic disturbances lie at the core of both the onset and progression of kidney diseases, establishing them as critical targets for therapeutic intervention. While current therapies targeting metabolic pathways show promise, further research and rigorous testing are necessary to determine their safety and efficacy in clinical settings. Additionally, other metabolic regulators may represent valuable targets to prevent the AKI-to-CKD transition, yet identifying the most effective and feasible targets requires extensive study. Interestingly, drugs currently used for unrelated conditions, such as diabetes management and chemotherapy-induced toxicity, are emerging as potential therapeutic options for kidney diseases. As our understanding of the intricate role of metabolism in kidney disease deepens, there is an urgent need for a paradigm shift in therapeutic strategies. A more refined understanding of the metabolic derangements underlying AKI and CKD can unlock new, targeted therapeutic opportunities that hold the potential to mitigate renal disease progression and improve patient outcomes.

## Data Availability

Not applicable.

## Abbreviations

acute kidney injury (AKI), chronic kidney disease (CKD), adenosine triphosphate (ATP), tricarboxylic acid (TCA) cycle, fatty acid oxidation (FAO), nicotinamide-adenine dinucleotide (NADH), flavin adenine dinucleotide (FADH_2_), proximal tubular epithelial cells (PTECs), cluster of differentiation 36 (CD36), carnitine palmitoyl-transferase 1 (CPT1), peroxisome proliferator-activated receptors (PPARs), PPARγ coactivator-1a (PGC1α), AMP-activated protein kinase (AMPK), acetyl-CoA carboxylase (ACC), glomerular filtration rate (GFR), Kruppel-like factor (KLF), hypoxia-inducible factor-1α (HIF-1α), phosphatase and tensin homolog (PTEN), phosphofructokinase (PFKL), pyruvate kinase M (PKM), sodium-glucose cotransporter 2 inhibitors (SGLT2is), endurance exercise training (EET), 5-aminoimidazole-4-carboxamide-1-*β*-D-ribofuranoside (AICAR).
